# Structural remodeling activates bacterial anti-phage immunity: insights from HerA-DUF4297

**DOI:** 10.3389/fcimb.2025.1612006

**Published:** 2025-06-12

**Authors:** Xiaoyan Wang, Leiliang Zhang

**Affiliations:** ^1^ Department of Clinical Laboratory Medicine, The First Affiliated Hospital of Shandong First Medical University and Shandong Provincial Qianfoshan Hospital, Jinan, Shandong, China; ^2^ Department of Pathogen Biology, School of Clinical and Basic Medical Sciences, Shandong First Medical University and Shandong Academy of Medical Sciences, Jinan, Shandong, China

**Keywords:** HerA-DUF4297, structural remodeling, phage, bacterial immunity, nuclease

## Abstract

In response to phage infection, bacteria have evolved a variety of sophisticated immune defense systems to combat viral predation. Among these defense mechanisms, the transmission of immune signals via intracellular signal transduction molecules is a common strategy that often accompanies enzyme activity. Recent studies have characterized the HerA-DUF4297 protein complex, a two-component defense system that integrates ATPase and nuclease activities. This complex inhibits phage infection by inducing DNA degradation. Notably, DUF4297 displays minimal nuclease activity when it operates on its own. However, it demonstrates robust nuclease activity when in complex with HerA. Crucially, the nuclease activity within this complex is regulated by structural changes. These findings provide novel insights into the activation of bacterial immune systems against phages, suggesting that the architectural remodeling of protein complexes can serve as a mechanism for transmitting immune signals.

## 1 Introduction

During the long process of evolution, bacteria have developed various defense mechanisms against phage infection. Many defense systems coupled with enzyme activity inhibit phage proliferation by degrading nucleic acids and/or essential intracellular metabolites. The activation of enzyme activity can be achieved by recognizing conserved viral proteins and sensing changes in intracellular signal transduction molecules. For instance, Avs proteins form Avs tetramers by recognizing the characteristic proteins produced during phage infection, thereby activating the N-terminal nuclease activity to degrade phage nucleic acids (Gao et al., 2022). Compared with regulating enzyme activity by recognizing virus specific proteins, it is more common to regulate enzyme activity through intracellular signal transduction molecules. For example, in the Gabija system, the enzyme activity of GajA protein is affected by sensing changes in nucleotide concentration (Cheng et al., 2021). In Type III CRISPR, CBASS, Pycsar and Thoeris defense systems, the enzyme activity is regulated by the second messenger cyclic nucleotide (Athukoralage and White, 2022; Wang and Zhang, 2023; Hobbs and Kranzusch, 2024).

**Figure 1:**
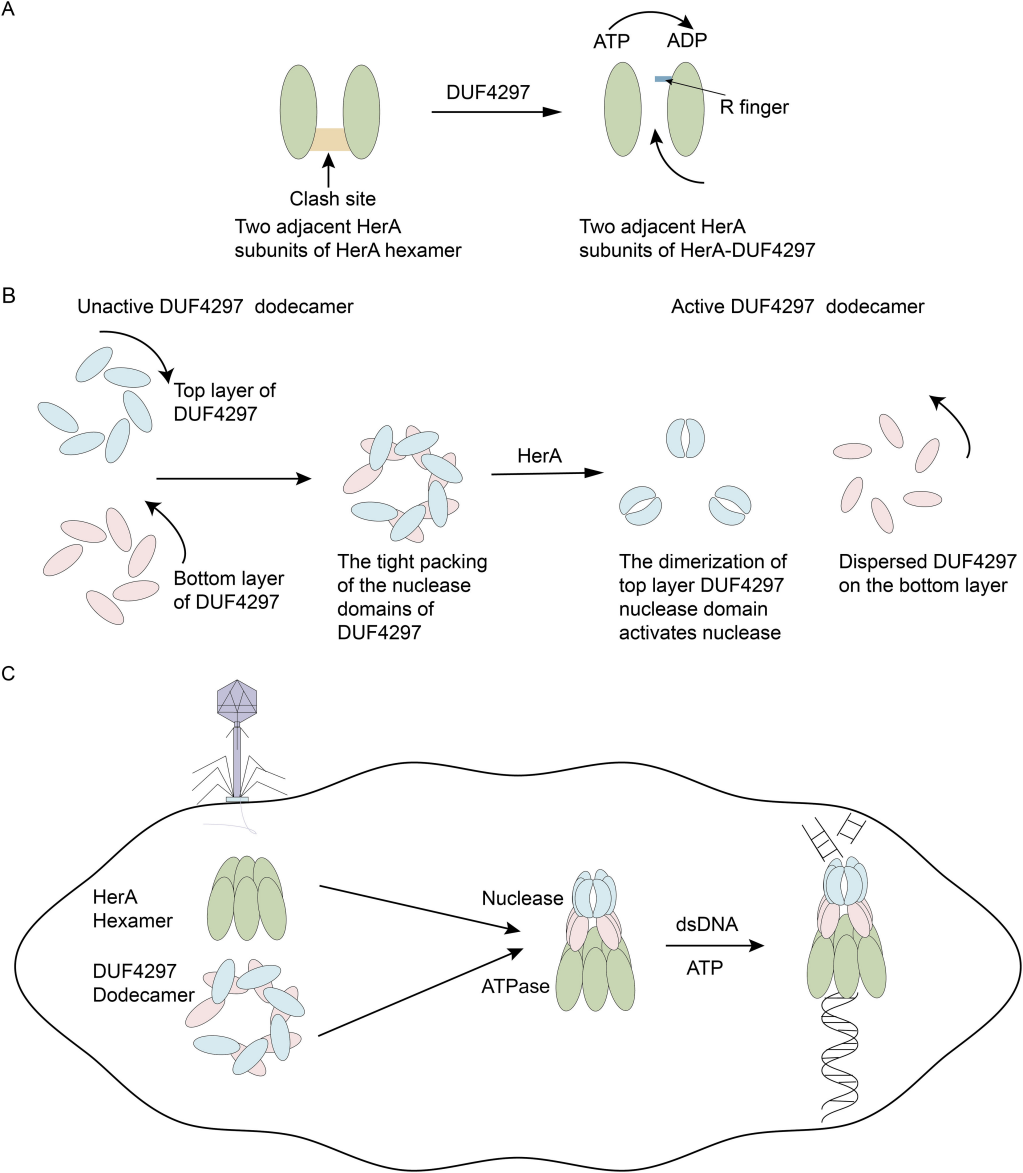
Conformation remodeling of HerA-DUF4297 activates their enzymatic activities. **(A)** Upon forming a complex with DUF4297, the R finger relocates to hydrolyze ATP. The clash site created by the insertion and helix bundle domains is colored orange. **(B)** Upon forming a complex with HerA, the upper DUF4297 molecules adopt a clamp-shaped dimer conformation, while the lower DUF4297 molecules display a more dispersed structure. **(C)** Phage infection initiates the assembly of HerA and DUF4297 into a complex, leading to the remodeling of the complex, which in turn activates their enzymatic activities.

In recent studies, the biochemical characterization of a two-component defense system HerA-DUF4297 that couples enzyme activity provide new insights into the activation of bacterial anti-phage immune systems (An et al., 2024; Rish et al., 2025; Tang et al., 2025). HerA possesses ATPase activity. DUF4297 has a Cap4-like nuclease domain (**Figure 1A**). HerA and DUF4297 assemble into a ~1-MDa octadecameric complex (An et al., 2024; Tang et al., 2025), with 12 DUF4297 molecules forming two layers on top of the HerA hexamer, each layer consisting of six DUF4297 molecules (An et al., 2024; Rish et al., 2025; Tang et al., 2025) (**Figure 1B**). Interestingly, the assembly of the complex induces dimerization of the upper layer DUF4297 molecules, and HerA transforms into a symmetrical planar hexamer (An et al., 2024; Rish et al., 2025) (**Figure 1C**). The assembled complex exhibits strong nuclease and ATPase activities (An et al., 2024; Rish et al., 2025; Tang et al., 2025). This suggests that protein complex architectural remodeling can serve as an immune signal for bacterial anti-phage defense, providing a new perspective for studying the activation of bacterial anti-phage immune systems.

## 2 Spatial structure of HerA and DUF4297

HerA possesses ATPase activity and is composed of three domains: the HAS domain at the N-terminus, the RecA-like catalytic center, and the Helix bundle domain, followed by the C-terminal hook. An et al. found that compared with the thermophilic archaeon *Sulfolobus solfataricus* HerA (SsoHerA), HerA has an additional insertion in the helix bundle domain and C-terminal hook (An et al., 2024). Moreover, in the bacterial anti-phage immune Sir2-HerA defense system, no similar C-terminal hook structure was observed (Liao et al., 2024). Cryo-electron microscopy (cryo-EM) revealed that HerA exists as an asymmetric hexamer alone, with the bottom of the HerA hexamer presenting a completely closed conformation (Tang et al., 2025). Additionally, there is a gap (cleft) between the top and bottom subunits, causing HerA to adopt a split helical conformation. HerA in this conformation does not exhibit the same ATPase activity as the HerA-DUF4297 complex (An et al., 2024). The HerA hexamer exhibited low ATPase activity because the insertion and helix bundle domains hinder the relocation of the R finger, which is necessary for ATP hydrolysis (An et al., 2024).

DUF4297 contains an N-terminal domain (NTD) and a C-terminal domain (CTD), with the NTD containing a Cap4-like nuclease domain but not showing nuclease activity itself. When DUF4297 exists alone, it displays multiple oligomers. Rish et al. found through structural analysis that dodecamers are the dominant species (Rish et al., 2025). DUF4297 dodecamers are composed of two layers, with each layer forming an open-ring structure from six protomers. The 12 CTDs form a cylindrical core, and the nuclease domains are arranged on the sides.

## 3 Assembly of HerA-DUF4297 complex

Cryo-EM further revealed the detailed assembly process of the HerA-DUF4297 complex. Twelve DUF4297 molecules form an octadecamer complex with the top of the HerA hexamer in two layers, with six molecules in each layer. The six DUF4297 molecules in the top layer are mainly held together by salt bridges and hydrogen bonds mediated by the CTD domain. The DUF4297 molecules in the bottom layer form tail-to-tail dimers with the top layer DUF4297 molecules through loops connecting helices α3 and α4, with hydrophobic interactions dominating at this interface (An et al., 2024). Unlike the isolated HerA hexamer, the complex shows a symmetrical planar hexamer of HerA with a central channel. The DUF4297 molecules in the bottom layer interact with the HAS domains of two adjacent HerA molecules through the CTD domain, and this interface is mainly mediated by hydrogen bonds. The C-terminal hook structure of HerA strengthens the connection between adjacent subunits. Meanwhile, the three domains of HerA extensively contact the same domains of adjacent subunits, further enhancing the interaction between the bottom layer HerA molecules. The HAS domains of the bottom layer HerA molecules form an approximately 6-fold symmetry with DUF4297, and the formed complex has a central channel.

## 4 Remodeling of HerA-DUF4297 regulates its activities

Unlike DUF4297 alone, the HerA-DUF4297 complex exhibited strong nuclease activity (An et al., 2024; Rish et al., 2025; Tang et al., 2025). Similarly, while HerA alone demonstrated very low ATPase activity, its formation of a complex with DUF4297 resulted in significantly enhanced ATPase activity. Rish et al. mutated HerA residues that play an important role in complex formation, including E92K, E25K and H44D, the mutant complex showed lower nuclease activity (Rish et al., 2025), suggesting that HerA’s interaction with DUF4297 regulates their activities.

In the complex, it was observed that HerA forms a symmetrical structure, has more extensive interactions with adjacent subunits, the helical domains form narrower pores, and the HAS domain forms wider pores. These structural changes may facilitate the coordination between the relocation of the R finger and the insertion and helix bundle domains, ensuring the R finger correctly positions itself. Subsequently, the R finger contributes to the formation of the ATPase active site, resulting in HerA exhibiting strong ATPase activity (**Figure 1A**). In the isolated DUF4297 dodecamers, the top and bottom DUF4297 molecules are closely connected in a clockwise and counterclockwise direction, respectively, which leads to the masking of the nuclease domain. When forming a complex, the HAS domain of HerA interacts with the CTD domain of the bottom DUF4297 molecule, causing the bottom DUF4297 molecule to tilt at a certain angle and adopt a more dispersed conformation, extending the conformation of the nuclease domain (Rish et al., 2025). The alteration of the bottom structure results in significant conformational changes in the nuclease domain and CTD domain of the adjacent top DUF4297 molecules, forming a clamp-shaped dimer (**Figure 1B**). An et al. and Rish et al. mutated the residues mediating the formation of DUF4297 dimers and found that the nuclease activity of the complex was significantly reduced (An et al., 2024; Rish et al., 2025), indicating that the dimerization of the nuclease domain is crucial for the activation of DUF4297 nuclease. After the complex formation, double-stranded DNA (dsDNA) enters the complex through the pore of the HerA hexamer, and HerA provides energy for DNA transport by hydrolyzing ATP, and is finally degraded by the nuclease aggregation region, inducing cell death. In summary, the conformational changes in the complex caused by the binding of HerA lead to the activation of enzyme activity, thereby triggering the activation of the bacterial anti-phage immune system (**Figure 1C**).

## 5 Enlightenments of HerA-DUF4297 for two-component defense systems coupled with both the nuclease and ATPase activities

In the HerA-DUF4297 defense system, the structural changes of the complex activate the enzymatic activity. In recent years, several two-component systems with potential nuclease and ATPase activities have been characterized (**Table 1**) (Sasaki et al., 2023; Tang et al., 2023; An et al., 2024; Li et al., 2024; Tuck et al., 2024; Zhen et al., 2024; Rish et al., 2025; Tang et al., 2025). In the newly characterized two-component defense system HamAB (DUF1837-HamB), the HamB domain rotates upon substrate recognition, disrupting the interaction interface with HamA and leading to the activation of HamA’s nuclease activity (Tuck et al., 2024). In another recently widely studied defense system, Sir2-HerA demonstrates various enzymatic functions, including nuclease and ATPase activities, by assembling into an octadecomer complex that degrades phage genes (Tang et al., 2023). Sir2 forms a dodecamer with two layers, each layer consisting of six molecules. HerA binds to the bottom layer of Sir2 in a hexamer form. Interestingly, Zhen et al. found that in *Staphylococcus aureus*, the Sir2-HerA (SaSir2-HerA), the binding of SaHerA alters the α15 helix structure of Sir2, opening the NAD+ binding pocket of Sir2 to achieve NADase function (Zhen et al., 2024). This highlights the importance of structural changes in the activation of two-component defense systems, suggesting that the regulation of enzymatic activity through complex conformational changes may be applicable to other similar systems.

**Table 1:** The potential two-component defense systems with both nuclease and ATPase activities.

Two-component defense system	ATPase domain	Nuclease domain	Whether to form a complex or not	The activation mechanism of nuclease	Speculated anti-phage mechanism	References
HerA-DUF4297	HerA	DUF4297	Yes	The structural remodeling of HerA-DUF 4297 induces the dimerization of the top-layer DUF 4297 nuclease domain, thereby activating its nuclease activity.	Structural remodeling activates enzyme activities that help resist phage infection through abortive infection.	(An et al., 2024; Rish et al., 2025; Tang et al., 2025)
HamAB	HamB	HamA	Yes	The conformational change induced by HamB’s recognition of the 3’ ssDNA ends triggers the activation of HamA.	The activated HamAB resists phage infection through abortive infection.	(Tuck et al., 2024)
Sir2-HerA	HerA	Sir2	Yes	Unclear	By assembling into an octadecamer complex, it degrades phage genes.	(Tang et al., 2023; Zhen et al., 2024)
PtuAB	PtuA	PtuB	Yes	Unclear	The oligomerization of PtuA forms a hexamer, which recruits two PtuB molecules to create the PtuAB complex. This activated complex can selectively degrade phage DNA.	(Li et al., 2024)
AbpAB	AbpB	AbpA	No	By sensing the phage-encoded ssDNA-binding protein or those formed by interrupting host DNA replication or repair	The activated AbpAB resists phage infection through abortive infection.	(Sasaki et al., 2023)

## 6 Conclusions and prospects

Through the structural analysis of the HerA-DUF4297 complex, researchers have observed the role of structural remodeling in activating the bacterial anti-phage immune response. Both HerA and DUF4297 lack the corresponding enzymatic activity when they are alone. However, upon forming a complex, their interaction causes HerA to transition from a non-planar structure to a planar hexamer. This conformational change facilitates the dimerization of the upper DUF4297 molecules, thereby activating their respective enzymatic activities. Notably, this dimerization structural change is similar to that of the AVAST3 (antiviral ATPases/NTPases of the STAND superfamily type 3) nuclease domain (Gao et al., 2022). When the Avs (antiviral STAND) protein recognizes phage proteins, the STAND ATPase domain mediates the dimerization of the N-terminal nuclease domain of Avs3, leading to the activation of the nuclease (Gao et al., 2022). It is speculated that inducing the formation of adjacent dimers might be a universal activation mechanism.

After phage infection, the expression of HerA and DUF4297 is induced, but the specific triggering mechanism remains unclear. dsDNA is transported through the central channel of the complex to the nuclease aggregation region for degradation. However, the complex lacks specificity in degrading dsDNA, which also suggests that the expression of HerA and DUF4297 in bacteria not infected by phages is strictly negatively regulated. In response to the abortive infection mediated by the HerA-DUF4297 complex, phages can produce the JSSI-004 protein to phosphorylate the HerA-DUF4297 complex to achieve immune escape (Jiang et al., 2024).

The immune battle between bacteria and phages never ceases. From the HerA-DUF4297 complex, we gain insight into the intriguing mechanism of structural remodeling that activates the immune defense system. This offers a new perspective for exploring unknown two-component defense systems and potentially more complex immune systems.
